# Recent Advances in Collagen Antimicrobial Biomaterials for Tissue Engineering Applications: A Review

**DOI:** 10.3390/ijms24097808

**Published:** 2023-04-25

**Authors:** Caglar Ersanli, Athina Tzora, Ioannis Skoufos, Chrysoula (Chrysa) Voidarou, Dimitrios I. Zeugolis

**Affiliations:** 1 Laboratory of Animal Science, Nutrition and Biotechnology, Department of Agriculture, University of Ioannina, 47100 Arta, Greece; c.ersanli@uoi.gr; 2 Laboratory of Animal Health, Food Hygiene and Quality, Department of Agriculture, University of Ioannina, 47100 Arta, Greece; tzora@uoi.gr (A.T.);; 3 Regenerative, Modular & Developmental Engineering Laboratory (REMODEL), Charles Institute of Dermatology, Conway Institute of Biomolecular and Biomedical Research, School of Mechanical and Materials Engineering, University College Dublin, D04 V1W8 Dublin, Ireland

**Keywords:** collagen, microbial infection, antimicrobial resistance, biomaterial-based therapies, antibacterial activity, tissue regeneration

## Abstract

Biomaterial-based therapies have been receiving attention for treating microbial infections mainly to overcome the increasing number of drug-resistant bacterial strains and off-target impacts of therapeutic agents by conventional strategies. A fibrous, non-soluble protein, collagen, is one of the most studied biopolymers for the development of antimicrobial biomaterials owing to its superior physicochemical, biomechanical, and biological properties. In this study, we reviewed the different approaches used to develop collagen-based antimicrobial devices, such as non-pharmacological, antibiotic, metal oxide, antimicrobial peptide, herbal extract-based, and combination approaches, with a particular focus on preclinical studies that have been published in the last decade.

## 1. Introduction

Microbial infections threaten public health due to the wide range of enervating effects of disease-causing microbes (e.g., bacteria, viruses, and fungi), which have been the primary causatives of the dissemination of pathogenic diseases [1,2,3]. Antibiotics have been the first choice for infection treatment since the discovery of penicillin in 1928 by Alexander Fleming. Although their low toxicity and great bactericidal features, the usage of antibiotics for a long time led to the burst and release of antibiotic-resistant bacteria (ARB), hence the emergence of antimicrobial resistance (AMR) related diseases [4,5].

Healthcare-associated infections are the major type of AMR-caused infections that may delay discharge from the hospital or cause deaths as well as a rise in healthcare costs, second-line drug costs, and unsuccess in treatments [6]. According to the European Centre for Disease Prevention and Control (ECDC), in Europe, annually, 3.8 million people catch healthcare-associated diseases caused by AMR [7], and 90 thousand people die because of these diseases [8]. Besides, the Centre for Disease Control and Prevention (CDC) reported that more than 2.8 million people suffer from AMR diseases each year, while 35,000 patients die in the US [9]. Moreover, the cost for just one AMR infection case is predicted approximately EUR 9–34 thousand more than non-resistant microbial infections [10], whilst more than EUR 9 billion are required in Europe [11,12]. On the other hand, bacterial resistance itself adds more than USD 20 billion to healthcare costs in the US [9].

In response, a variety of clinical interventions have been employed to combat AMR-related diseases, including the use of combination therapies, strategies aim targeting antimicrobial-resistant enzymes or bacteria, longer treatment durations, and off-label uses [13,14]. Despite these efforts, the development of new and effective antimicrobials has been slow, and the emergence of resistance to these interventions has become incremental. Furthermore, some of these interventions come with their drawbacks, such as an increase in side effects, higher medicinal costs, and longer hospital stays [15]. As such, there is a clinical need for alternative approaches such as biomaterial-based strategies to combat antimicrobial resistance and promote the development of more effective therapies. The various antimicrobial collagen biomaterial strategies have recently come to the fore in the literature within this framework.

**Figure 1:**
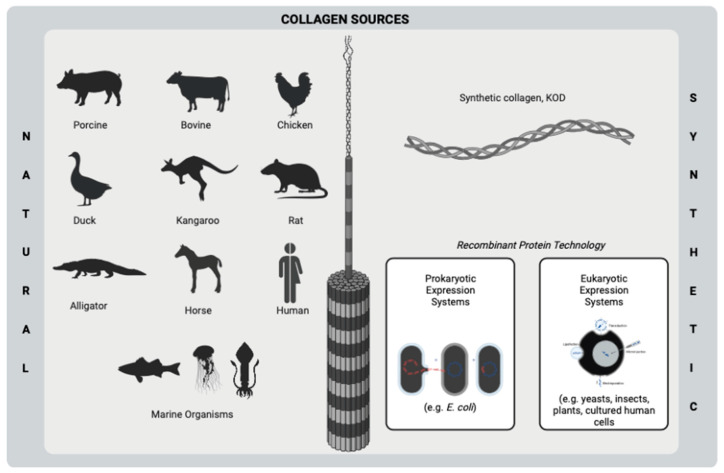
Sources of collagen using biomedical purposes. This figure was created by BioRender.com.

Collagen is a fibrous, insoluble protein that is the main component of the extracellular matrix (ECM) of several tissues [16] of humans and many animals, such as bone [17], cartilage [18], tendon [19], skin [20], and muscle [21]. A natural biopolymer, collagen is one of the most abundant proteins in mammals [22] and becomes prominent among the other polymers due to its superior and distinct properties. Excellent biocompatibility, good biodegradability, hydrophilicity, remarkable mechanical properties, low or no antigenicity, hemostatic properties, and cell-binding ability are some of the important features of collagen, which make it important for many biomaterial applications, such as tissue engineering and drug delivery purposes [23,24,25,26]. On the other hand, the resistance of collagen to bacteria makes it outstanding to use in the development of antimicrobial biomaterials for many kinds of applications, such as the treatment of wounds and bone infections. Owing to its natural ability to fight infection, collagen contributes to keeping the infection site sterile [26,27]. Moreover, collagen has very high availability since its abundance in mammals and marine organisms as well as its producibility from yeasts, plants, insects, and mammal cells by recombinant protein technology [23,28] (Figure 1). Despite its proven properties, collagen-based biomaterials need to incorporate bioactive molecules such as antibiotics and plant-based agents in order to increase their biological activities.

**Figure 2:**
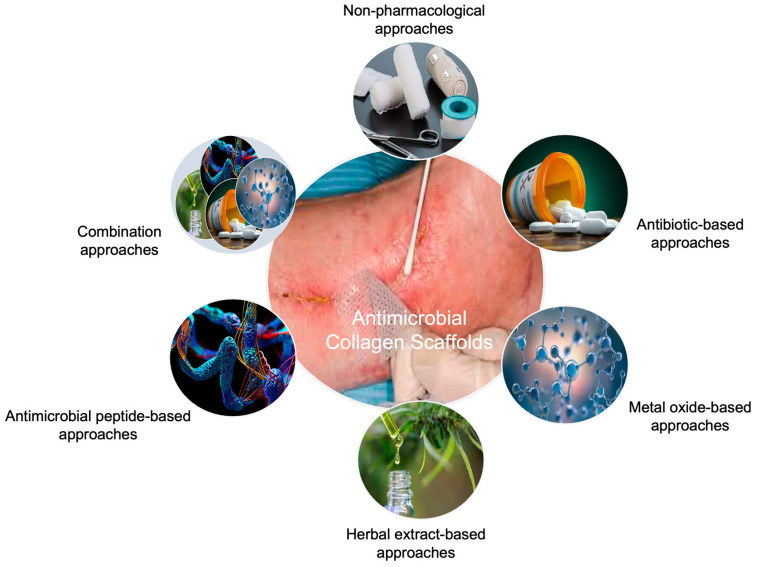
Approaches to developing collagen-based antimicrobial biomaterials for tissue engineering applications.

The use of alternative antimicrobial agents (e.g., herbal extracts, antimicrobial peptides, and metal oxide nanoparticles) as a substitute for antibiotics has started to gain importance in an attempt to overcome the emergence of AMR due to ARB strains [2,29,30]. Even though clinically proven, generally, single and limited antibacterial agents (e.g., silver and gentamicin), including collagen-based products, are on the market (Table 1). Therefore, new modern products are clinically needed to improve treatment efficacy. In this respect, collagen has been widely used as a carrier vehicle for several kinds of bioactive molecules with their ensured biostability owing to its superior biological activities [23,24,25]. Herein, we briefly reviewed different approaches for designing collagen-based antimicrobial products (Figure 2) with a particular focus on preclinical studies which have been published in the last decade.

## 2. Non-Pharmacological Approaches

In the literature, there are several studies concerning collagen-based biomaterial therapies for healing microbial infections without the incorporation of any therapeutics (Table 2). Chitosan is a commonly used additive polymer for collagen scaffolds to enhance bactericidal effects due to its good antibacterial activity against several Gram-positive and Gram-negative bacterial strains [47]. Chitosan and oxidized bacterial cellulose with composite collagen hemostasis dressings exhibited a faster hemostasis rate (86 s) than commercial gauze (186 s) in vivo rat liver trauma model through the collagen to promote platelet and erythrocyte adhesion as well as to improve pro coagulation activity [48]. Collagen hydrolysate wound dressings, including chitosan and tetraethoxysilane (TEOS), accelerated the healing of wounds in the Wistar rats compared to gauze, where the wound recovered completely within 14 days. Besides the augmented healing process, the re-epithelization rate was evaluated as 81% and 55% for composite and control groups, respectively on the 10th post-treatment day. However, despite the successful preclinical findings, developed dressings could not inhibit the growth of *P. aeruginosa*, which is one of the most common causative bacteria for wound infections [49]. On the other hand, some inorganic compounds were incorporated in biomaterial formation to increase targeted tissue regeneration and antimicrobial activity [50,51,52]. For instance, the association of collagen with bioactive glass (BG) may promote the antibacterial activity of pristine collagen by the increase in osmotic pressure, which is raised proportionally to the released ions (e.g., silicon, calcium, and phosphorous) composed of bioactive glasses. Hence, the growth of bacteria is inhibited because of the formed region by ions. It is reported that collagen/BG scaffolds implanted in Sprague–Dawley (SD) rats’ dorsum skin defect healed the wound faster than the clinically used product, Kaltostat, and triggered re-epithelization regarding histologic results [52].

## 3. Pharmacological Approaches

### 3.1. Antibiotic-Based Approaches

Antibiotics are well-known antimicrobial drugs that have an important role in the treatment of bacterial infections by fighting and preventing the growth of bacteria [60]. The incorporation of various antibiotics, such as aminoglycosides [61,62,63,64] and tetracyclines [65,66,67,68,69], into collagen scaffolds have been studied for a long time (Table 3). These therapeutic agents are generally studied for infected wound and bone defect treatments. For example, when mupirocin was loaded into collagen sponges, complete closure and re-epithelization on full-thickness excision wounds treated with the developed composite scaffold were achieved. Nevertheless, scaffolds could provide significant antibacterial activity against Gram-positive methicillin-resistant *S. aureus* (MRSA) and *B. subtilis* [70]. In another study of a commonly studied antibiotic, doxycycline-loaded collagen-based scaffolds increased the gap closure of bone defects in Wistar rats from 25% to 40% [69]. On the other hand, the concentration of collagen is an effective parameter for the controlled antibiotic release from a biomaterial. An increase in collagen concentration from 20% to 40% (*w*/*w*) did not enhance the in vivo healing of mice wounds, treated with cefazolin, including collagen-based nanofibrous mat, due to inadequate release of antibiotics on the wound bed. This outcome indicates the role of polymer concentration in the sustained release of an incorporated antimicrobial agent in a biomaterial formulation [71].

In some strategies, the effect of antibiotics is enhanced by the addition of chitosan into the biomaterial formulation. Chitosan exerts its antibacterial activity by binding to the negatively charged bacterial cell wall, thus initiating a process that leads either to the disruption of bacterial cells or to a change in the bacterial membrane permeability [72]. For instance, minocycline-caged chitosan nanoparticles incorporated into collagen sponges demonstrated almost complete degradation and no remarkable inflammation in the SD rat skull defect model [66]. The antimicrobial activity of biomaterials was also advanced by generating a hypoxic environment by including oxygen-generating additives. Oxygen-generating calcium peroxide particles were coated on the ciprofloxacin-loaded collagen-based sponges by Tripathi et al. to advance the wound healing rate by generating a hypoxic environment. The tested scaffolds on the skin flip model led to less necrosis and displayed almost total wound recovery with the help of the antibacterial activity of the antibiotic and hypoxic conditions, whilst the untreated group showed about 75% of wound closure within 15 days [73]. Even though good inhibitory effects are reported, it is known that long-term use of antibiotics results in the emergence of ARB strains. In the attempt to research a few alternatives, non-antibiotic therapeutic approaches have become inevitable.

**Table 3:** Illustrative examples of collagen antimicrobial scaffolds designed with antibiotic-based approaches. Abbreviations: Antimicrobial susceptibility testing: AST; Weight: *w*; Volume: *v*; Collagen: Col; Chitosan: Chi; Hyaluronic acid: HA; Hexamethylene diisocyanate: HMDI; Cefaclor: Cef; Ranalexin: Ran; *Escherichia coli*: *E. coli*; *Staphylococcus epidermidis*: *S. epidermidis*; *Staphylococcus aureus*: *S. aureus*; *Pseudomonas aeruginosa*: *P. aeruginosa*; *Enterococcus faecalis*: *E. faecalis*; Methicillin-resistant *Staphylococcus aureus*: MRSA; *Bacillus subtilis*: *B. subtilis*; *Porphyromonas gingivalis*: *P. gingivalis*; *Fusobacterium nucleatum*: *F. nucleatum*; Hydroxy apatite: Hap; Citrate hydroxy apatite: cHap; N-(3-dimethylaminopropyl)-N-ethylcarbodiimide hydrochloride: EDC; N-hydroxysuccinimide: NHS; Nanoparticle: NP; Sodium tripolyphosphate: TPP; Poly(lactic acid): PLA; Sodium hydroxide: NaOH; Ethanol: EtOH; Polyethylene oxide: PEO; Polycaprolactone: PCL; Ethyl cellulose: EC; Polyvinylpyrrolidone: PVP; Bioactive glass: BG; Glutaraldehyde: GTA; Human bone marrow-derived stromal cell culture: hBM-MSC; Calcium peroxide: CPO; Cellulose nanocrystal: CNC; Ciprofloxacin hydrochloride: CFH; Rabbit adipose-derived stem cell: rASC.

Composition	Collagen Source	Scaffold Form	Crosslinking	Therapeutic Agent	Release Profile	AST	Bacterial Strain/ Cell Line	Antibacterial Activity	Hypothetic Material	Refs.
Col (5% *w*/*v*)	Porcine dermis	Sponge	HMDI (0.625–10% *w*/*v*)	Cef, Ran (0–500 µg/mL)	10 µg/mL of 90% Cef and 95% Ran released by day 7.	Disc diffusion	*E. coli*, *S. epidermidis* Adult HDFs	100 µg/mL of Cef showed activity on tested strains, while Ran did not.	Localized drug delivery vehicle	[74]
Col (1% *w*/*w*) Chi (1% *w*/*w*) HA (1% *w*/*w*)	Rat tail tendon	Thin film	Not available	Gentamicin sulfate (0.4 mg/cm^2^ film)	Not studied	Disc diffusion	*S. aureus*, *E. coli*, *P. aeruginosa* No in vitro cell culture	Drug-loaded scaffolds showed approximately 25–30 mm of inhibition zone.	Antibacterial film	[61]
Col (8 *w*%) Hap (0–15 *w*%)	Type I (not specified)	Micro/nanostructured layers	EDC/NHS (4:1 *w* ratio)	Vancomycin hydrochloride (10 *w*% of Col)	The max released concentration of vancomycin exceeded the MIC by up to 60–75 times for 4 weeks.	Disc diffusion	MRSA, *S. epidermidis*, *E. faecalis* SAOS-2 osteosarcoma cells	Inhibition zone diameters did not differ from standard antibiotic discs significantly.	Local drug carrier	[62]
Col (3 mg/mL)	Bovine tendon	Sponge	Not available	Mupirocin (2 mg/mL) (caged into silica microspheres)	Almost 90% of mupirocin was released within 3 days from sponges.	Broth dilution	*B. subtilis*, *S. aureus*, *E. coli*, *P. aeruginosa* 3T3-L1 fibroblasts	Drug-loaded wound dressings did not show sufficient antibacterial activity on *B. subtilis* and *E. coli*.	Wound dressing	[70]
Col Chi (2% *w*/*v*)	Mouse tail tendon	Asymmetric membrane	TPP (0.2% *w*/*v*)	Minocycline (15 µg/mL) (caged into Chi NPs)	Minocycline had sustained release until the 7th day.	Live/dead bacterial double staining	*P. gingivalis*, *F. nucleatum* MC3T3-E1 osteoblasts, L929 fibroblasts	Membranes showed 95.3%, and 92.1% of bacteriostatic activity against *P. gingivalis* and *F. nucleatum*, respectively.	Scaffold for the prevention of infection and guide bone regeneration	[65]
Col hydrolysate (5 mg/mL) PLA (5 mg/mL) cHap (10 mg)	Type I (not specified)	3D-printed porous scaffold	Alkali hydrolysis (1:1 NaOH:EtOH and 0.5% *w*/*v* citric acid)	Minocycline hydrochloride (0.5 mg/mL)	A burst release of minocycline was observed within the first hour.	Disc diffusion, biofilm inhibition assay	*S. aureus* hBM-MSCs	Drug-loaded scaffolds showed smaller inhibition zone than standard antibiotic discs.	Antimicrobial and osteogenic scaffold	[66]
Col (10 *w*%) PLA EC (7:3, 8:2, 9:1 EC/PLA *w* ratio)	Fish collagen	Nanofibrous mat	Not available	Silver sulfadiazine (0.25, 0.5, 0.75 *w*%)	28 ppm of silver ions were released from 0.75 *w*% drug-loaded mats within 96 h.	Disc diffusion	*Bacillus*, *E. coli* NIH 3T3 fibroblasts	Only 0.75% of drugs including scaffolds showed antibacterial activity against tested strains.	Wound dressing	[75]
Col (10, 20, 40% *w*/*w*) PVP (30% *w*/*v*) PLA PEO (Shell–Col/PVP, Core: 80:20 PLA/PEO *w*/*w*)	Bovine tendon	Nanofibrous mat	Not available	Cefazolin sodium	44.15%, 40.80%, and 37.76% of cefazolin were released for samples containing 10%, 20%, and 30% (*w*/*w*) collagen after 6 days.	Disc diffusion	MRSA, *E. coli*, *P. aeruginosa* No in vitro cell culture	Fabricated mats showed slightly higher antibacterial activity against *P. aeruginosa*.	Antibacterial patch for wound healing	[71]
Col (10 mg/mL)	Fish collagen	Hydrogel	Alginate dialdehyde (2–10 mg/mL)	Tetracycline hydrochloride (0.01–0.2 mg/mL)	Almost 20% of antibiotics with a concentration equal to or higher than 0.1 mg/mL were released during 600 min.	Zone inhibition	*S. aureus* 3T3 fibroblasts	Dressings did not show high inhibition rates of *S. aureus*.	Wound dressing	[67]
Col BG (0.5 mg/mL)	Bovine	Membrane (Commercial product)	Not available	Tetracycline hydrochloride (0.05, 0.2, 0.35 mg/mL)	More than 50% of tetracycline releases within the first 6 h, and significant release was observed in 24 h.	Zone inhibition Plate counting	*S. aureus* (different strains), *S. epidermidis* MG-63 osteosarcoma cells	Developed scaffolds could significantly inhibit *S. aureus* growth.	Scaffold for the prevention of biomaterial-related infections	[68]
Col (1.5% *w*/*v*) Chi (1.5% *w*/*v*) CPO (1–4 *w*%)	Bovine	Sponge	EDC/NHS (3 *w*%, 2:1 EDC:NHS *w* ratio)	Ciprofloxacin hydrochloride (1 mg/mL)	Almost 80% of CFH was released from scaffolds including 4% CPO within 200 h.	Zone inhibition	*E. coli*, *S. aureus* HDFs	Scaffolds displayed good inhibition zones against both strains.	Skin tissue engineering scaffold	[73]
Col (5% *w*/*v*) Hap (10 *w*%)	Rat tail	Sponge	EDC (0.1 mM)	Doxycycline containing Hap NPs (10 *w*%)	A sustained release of doxycycline (about 70%) was achieved over 14 days.	Time-kill assay	*S. aureus*, *P. aeruginosa* BM-MSCs	Antibiotic addition significantly reduced the number of colonies within 24 h.	Bone tissue engineering scaffold	[69]
Col (1 *w*%) CNC (5 *w*%)	Bovine tendon	Sponge	GTA (0.25%)	Gentamicin sulfate (25 mg/mL) impregnated gelatin microspheres	Gentamicin was completely released after 144 h of the incubation period.	Disc diffusion	*E. coli*, *S. aureus* NIH-3T3 fibroblasts	Composite scaffolds showed higher antibacterial activity against *E. coli* than *S. aureus*.	Antibacterial skin scaffold	[63]
Col Chi (4, 8, 16% total polymer, various Col/Chi *w* ratio)	Fish	Sponge	Not available	Norfloxacin (1 *w*%)	An almost complete release of the drug was observed within 20 h.	Not studied	No in vitro cell culture	Not studied	Scaffold for skin regeneration	[76]
Col (6.5 mg/mL)	Bovine tendon	Film	EDC/NHS (1:1:6 *w* ratio EDC/NHS/Col-Tobramycin)	Tobramycin (15 mg/mL)	The burst release of tobramycin (40%) was observed within the first 4 h.	Plate counting	*S. aureus* Human corneal epithelial cells	Tobramycin-loaded films showed significantly higher inhibition than pristine films.	Scaffold for corneal repair	[64]
Col Na-Alginate Hap	Cowhide	Sponge	Genipin CaCl_2_ (10 *w*%)	Amoxicillin (0.5, 1, 2 mg/mL)	The long-term drug release effect was investigated.	Zone inhibition	*E. coli* rASCs	Scaffolds could effectively inhibit *E. coli* growth.	Composite scaffold for infected bone defects	[77]

### 3.2. Non-Antibiotic-Based Approaches

Antimicrobial peptides (AMPs), metal oxides, and herbal extracts are remarkably interesting alternative antimicrobial agents to overcome the crucial drawbacks of antibiotics such as the emergence of ARB strains and the difficulties to treat biofilm-forming bacterial infections (Table 4). Hereby, we discuss the effect of these agents on antimicrobial therapies when incorporated into collagen scaffolds.

#### 3.2.1. Metal Oxide-Based Approaches

In recent years, there has been a great interest in metal oxide nanoparticles (NPs) to enhance the antimicrobial properties of collagen-based scaffolds due to their great inhibitory effects against broad-spectrum bacteria (Table 4). They can exert their bactericidal effect by linking to bacterial cell walls via electrostatic interactions [78], hydrophobic forces [79], van der Waals forces [80], and/or ligand binding [81]. Silver NPs are the well-known and most studied NPs in preclinical and commercial antimicrobial devices. In one study, silver NPs included collagen nanofibers presented an enhanced healing rate and led to the deposition of more hydroxyproline and collagen on the wound site in the Wistar rat model [82], whereas silver NPs loaded collagen hydrogels contributed to the reduction of pro-inflammatory cytokine IL-6 and inflammatory cytokines CCL24, TIMP1, and sTNFR-2, which indicates the exerted anti-inflammatory properties of silver NPs on the subcutaneous mice model [83]. Similarly, in vivo, 10 ppm silver NPs comprised collagen/chitosan hydrogel applied in full-thickness skin defects in the SD rat model expedited the fibroblast migration by the advance in α-SMA, upregulated the related macrophage activation, and downregulated inflammatory mediators [84]. However, the addition of silver NPs into the collagen scaffolds has not always exhibited a significant impact on wound healing. For example, both silver-loaded and pristine collagen membranes did not show complete wound closure [85], as silver NPs comprised collagen sponges [86]. Besides silver, zinc oxide NPs are also extensively studied in collagen biomaterials, owing to their well-recognized antibacterial and anti-inflammatory properties. For instance, the zinc oxide quantum dots were implicated in collagen/PCL nanofibrous mats for skin regeneration purposes and served as a suitable wound dressing. Both 0.75% (*w*/*v*) zinc oxide quantum dots included, pristine mats showed partial wound closure on full-thickness mice wound model at 12-day post-treatment with a wound closure rate of about 90%. Although scaffolds loaded with zinc oxide quantum dots presented a good inhibitory effect against *S. aureus*, their comparison with pristine scaffolds was not reported [87].

#### 3.2.2. Antimicrobial Peptide-Based Approaches

Antimicrobial peptides are environmentally friendly, small molecular weight, amphiphilic, and polycationic proteins that are composed of less than fifty amino acids in their structure [88,89,90]. They can cause cell lysis via binding to intercellular targets of negatively charged cell membranes [91,92] and exert bactericidal activity by the modulation of the host immune system [93]. Despite their good antibacterial action, AMPs have some drawbacks, such as a short half-life (within hours) and high manufacturing costs [94]. Hence, in the literature, the incorporation of AMPs into collagen scaffolds has been less researched than the other therapeutic agents (Table 4). AMP Tet213 incorporated collagen-based sponge dressings demonstrated almost complete wound healing on *E. coli*- and *S. aureus*-infected wounds on SD rats within 14 days, similar to pristine sponges and commercial silver-including products, in contrast to gauze control. As a result of Sirius red staining, pristine, and Tet213, loaded dressings exhibited around 60% of collagen deposition, which might be contributed by the biocompatibility of collagen. Moreover, according to the samples taken from the SD rat model on day 4, *E. coli* was 1.8 × 10^7^ CFU for gauze control, whereas no bacterial colonies were observed on wounds treated with Tet213 dressings [95]. In addition, it was observed that AMPs GL13K [96] and LL37 [97,98] incorporation into the collagen scaffolds increased their antibacterial activity against Gram-negative *E. coli*.

#### 3.2.3. Herbal Extract-Based Approaches

Plants have been used for traditional remedies (e.g., bone defects and burn wounds) for centuries [99,100]. The bioactive phytochemicals of herbs, such as phenolic substances, essential oils, vitamins, and phytohormones, gain them tremendous features (e.g., antimicrobial, antifungal, anti-inflammatory, and antioxidant activity) and make them a rising star for antimicrobial therapies as greener and safer therapeutics [101,102,103,104,105,106]. Therefore, there have been a remarkable number of attempts in the literature to incorporate various herbal extracts, such as cinnamon [107], *Cissus quadrangularis* [108], and thymol [109], into collagen scaffolds to create an ideal and alternative antimicrobial biomaterial strategy for tissue regeneration purposes (Table 4). The addition of curcumin into collagen/cellulose nanocrystal sponge dressings advanced epithelization rate and dermal cell proliferation while providing complete wound closure on full-thickness burn wounds within 21 days. Moreover, they significantly decreased the level of cytokines IL-1β, IL-6, and TNF-α between the 10th and 21st days and inhibited the NF-κB activity due to the long and sustained release of curcumin with antibacterial, antioxidant, and anti-inflammatory characteristics [110]. Thanks to their complex chemical structure, in some studies, herbal extracts are used as a crosslinker for collagen formulations as well as an antimicrobial therapeutic agent. For example, wheatgrass was studied as both an antimicrobial agent and a green crosslinker for collagen aerogels. The study observed that 2% (*w*/*v*) of wheat grass incorporation increased the size reduction of collagen aerogel-treated wounds from 47% to 75% on the 9th post-treatment day and triggered the angiogenesis within 24 h of incubation of the chick embryo model [111]. The concentration of the loaded herbal extract is determined as an effective parameter from the perspective of preclinical studies. To illustrate, the addition of 0.08 g of *Melilotus officinalis* extract exhibited better re-epithelization than 0.04 and 0.02 g on day 18 post-treatment, whereas the 0.08 g extract with collagen-based multilayer nanofibrous mat increased the collagen density in vivo from 55% to 82% within 18 days [112].

**Table 4:** Illustrative examples of collagen antimicrobial scaffolds designed with non-antibiotic-based approaches. Abbreviations: Antimicrobial susceptibility testing: AST; Weight: *w*; Volume: *v*; Collagen: Col; Chitosan: Chi; Nanoparticle: NP; Fibronectin: FN; Chondroitin 4-sulfate: CS; 1,4-Butanediol diglycidyl ether: BDDGE; Histidine: His; Hydroxy apatite: Hap; N-(3-dimethylaminopropyl)-N-ethylcarbodiimide hydrochloride: EDC; N-hydroxysuccinimide: NHS; Polycaprolactone: PCL; Glutaraldehyde: GTA; Titanium dioxide: TiO_2_; Antimicrobial peptide: AMP; Hyaluronic acid: HA; Poly(L-lactide-co-ε-caprolactone): PLC; Cellulose nanocrystal: CNC; Gelatin: Gel; Gulmohar seed polysaccharide: GSP; Poly(vinyl alcohol): PVA; *Escherichia coli*: *E. coli*; *Staphylococcus epidermidis*: *S. epidermidis*; *Staphylococcus aureus*: *S. aureus*; *Pseudomonas aeruginosa*: *P. aeruginosa*; *Bacillus subtilis*: *B. subtilis*; *Bacillus cereus*: *B. cereus*; *Salmonella enterica*: *S. enterica*; *Pseudomonas putida*: *P. putida*; *Porphyromonas gingivalis*: *P. gingivalis*; *Fusobacterium nucleatum*: *F. nucleatum*; *Streptococcus gordonii*: *S. gordonii*; Methicillin-resistant *Staphylococcus aureus*: MRSA; Spontaneously immortalized, human keratinocyte line: HaCaT; Human bone marrow-derived stromal cell culture: hBM-MSC; Human dermal fibroblast: HDF.

Composition	Collagen Source	Scaffold Form	Crosslinking	Therapeutic Agent	Release Profile	AST	Bacterial Strain/ Cell Line	Antibacterial Activity	Hypothetic Material	Refs.
**Metal oxide-based approaches**										
Col Chi (Various Col/Chi *w*% ratio)	Goat tendon	Thin film	EDC/NHS (2:1 M ratio)	Silver NPs (0.5 *w*%)	Not studied	Growth inhibition	*E. coli*, *S. aureus* MG-63 osteosarcoma cells	Up to 37% and 27% of growth inhibition was observed against *E. coli* and *S. aureus*, respectively.	Composite bone tissue engineering scaffold	[113]
Col FN CS (10:1:3 × 10^−5^ g/g Col/CS/FN)	Bovine tendon	Sponge	GTA (2.5% *v*/*v*)	Silver NPs (1 × 10^−4^ g/g polymers)	Not studied	Disc diffusion	*F.nucleatum*, *P. gingivalis* Gingival fibroblasts	Hybrid sponges showed slightly higher antimicrobial activity against *F. nucleatum*.	Oral cavity lesion dressing	[114]
Col (8% *w*/*v*)	Fish collagen	Nanofibrous mat	GTA (50% *w*/*v*)	Silver NPs (0.2% *w*/*v*)	Cumulatively, almost 100% of silver ions were released within 25 h.	Microdilution, disc diffusion	*S. aureus*, *P. aeruginosa* No in vitro cell culture	Approximately 3.2 and 2.3 cm of inhibition zone diameter was observed after 48 h against *S. aureus* and *P. aeruginosa*, respectively.	Wound dressing	[82]
Col (10% *w*/*w*)	Porcine	Hydrogel	BDDGE	Silver NPs (0.2 µM)	Steady silver concentration was reached within 0.5 h of incubation	Growth inhibition and Time-kill assays	*S. aureus*, *S. epidermidis*, *E. coli*, *P. aeruginosa* Human epidermal keratinocytes, and dermal fibroblasts	Hybrid hydrogels could inhibit the growth of all tested bacteria.	Implantable anti-infective hybrid biomaterial	[83]
Col (5% *w*/*w*) His (0, 1, 2% *w*/*w*)	Porcine	Membrane	EDC/NHS (3.55 and 2.13 mg/g)	Silver NPs	Not studied	Disc diffusion, bacterial suspension	*P. aeruginosa*, *S. aureus* L929 fibroblasts	Developed membranes did not show sufficient antimicrobial activity against both tested strains.	Dressing for full-thickness burn wounds	[85]
Col Chi (9:1 Col/Chi *w* ratio)	Bovine tendon	Hydrogel	EDC/NHS	Silver NPs (0, 2, 5, 10, 20 ppm)	Not studied	Disc diffusion	*E. coli*, *S. aureus* Mouse embryo fibroblasts, HaCaTs	Developed wound dressings showed higher inhibition of *S. aureus* growth.	Wound dressing	[84]
Col Hap (various *w* ratio)	Fish scale	Membrane	Genipin (0.003 g)	Silver NPs (0.05 *w*%)	Not studied	Disc diffusion	*E. coli*, *S. aureus* MG-63 osteosarcoma cells	Scaffolds presented less inhibition zone compared to standard ampicillin discs.	Bone filler	[115]
Col (0.5% *w*/*w*)	Bovine tendon	Sponge	Dialdehyde xanthan gum (10 mg/mL)	Silver NPs (10 mg/mL)	Not studied	Disc diffusion, bacterial infiltration	*E. coli*, *S. aureus*, *P. aeruginosa* L929 fibroblasts	An increase in silver NP concentration resulted in an increased inhibition rate against tested strains.	Antibacterial wound dressing	[86]
Col Sago starch (1, 2, 3 µM)	Fish scale	Sponge	Not available	Sago starch capped silver NPs (1:1 *w* ratio to Col)	Not studied	Broth dilution	*S. aureus*, *E. coli* NIH-3T3 fibroblasts	A lower minimum inhibitory concentration was examined against *E. coli*.	Scaffold for tissue regeneration applications	[116]
Col (1% *w*/*w*) Dextran	Calf hide	Hydrogel	GTA (0.25% *v*/*v*)	Zinc oxide NPs	Not studied	Not studied	No in vitro cell culture	Not studied	Wound dressing	[117]
Col (0.7 *w*%)	Bovine	Hydrogel	GTA (1% *v*/*v*)	Zinc oxide NPs (2, 3, 5 *w*%)	Not studied	Disc diffusion	*S. aureus*, *E. coli* No in vitro cell culture	The inhibition zone diameter decreased with increasing zinc oxide concentration against *S. aureus*.	Wound dressing	[118]
Col PCL (1:2, 1:1, 2:1, 3:1 Col/PCL *w* ratio)	Type I (not specified)	Nanofibrous mat	Not available	Zinc oxide quantum dots (0–0.75% *w*/*v*)	Not studied	Plate counting	*E. coli*, *S. aureus* L929 fibroblasts, 3T3 fibroblasts	The number of living bacteria was significantly reduced by the addition of 0.75% of NPs.	Antibacterial wound dressing	[87]
Col (1% *w*/*w*)	Calf hide	Sponge	GTA (0.5 *w*%)	Zinc titanate	Not studied	Disc diffusion	*S. epidermidis*, *B. cereus*, *E. coli*, *S. enterica*, *P. putida* MG-63 osteosarcoma cells, 3T3 fibroblasts HaCaTs	The porous nanocomposites exerted higher antimicrobial activity against *S. epidermidis*.	Anti-infection biomaterial	[119]
Col (5 mg/mL) Chi (5 mg/mL)	Pig skin	Sponge	GTA (2.5% *w*/*w*)	TiO_2_ NPs (1–7%)	Not studied	Bacterial culture, SEM imaging	*S. aureus* Mouse fibroblasts, red blood cells	Increased TiO_2_ amount led to reduced *S. aureus* colonies on the surface of the scaffold.	Wound dressing	[120]
Col (3.47 *w*%) Chi (3 *w*%)	Bovine	Nanofibrous mat	GTA (1 *w*%)	Zinc oxide NPs (1:1:1 *w* ratio Col:Chi:Zinc oxide)	Not studied	Disc diffusion	*S. aureus* Hep-2 cells	Membranes showed 4–8 mm of inhibition zone diameter against *S. aureus*.	Scaffold for skin tissue regeneration	[121]
Col Chi (1:9 Col/Chi *w* ratio)	Not specified	Sponge	Dehydrothermal crosslink at 105 °C for 24 h	Zinc oxide NPs (1, 3, 5 *w*%)	Not studied	Disc diffusion	*E. coli*, *S. aureus* No in vitro cell culture	*S. aureus* was found more sensitive to developed scaffolds than *E. coli*.	Antibacterial product	[122]
**Antimicrobial peptide-based approaches**										
Col (3 mg/mL)	Bovine	Hydrogel	EDC/NHS (50 mM EDC, 25 mM NHS)	AMP GL13K (1 mM)	Burst release was observed from 21 to 28 days.	ATP bioluminescence, live/dead assays	*S. gordonii*, *E. coli* hBM-MSCs	AMP GL13K coating significantly demonstrated less effects on the membrane integrity of *S. gordonii*.	Scaffold for bone/dental tissue growth and infection prevention	[96]
Col (0.6% *w*/*v*) HA (0.5% *w*/*v*) Alginate (1.2% *w*/*v*)	Type I (not specified	Sponge	EDC/NHS (0.6 mg/mL EDC, 0.3 mg/mL NHS)	AMP Tet213 (500 µg/mL)	Sustained release (68.4 ± 10.2%) was observed after 14 days.	Zone inhibition, colony counting	*E. coli*, MRSA, *S. aureus* NIH-3T3 fibroblasts	The addition of AMP Tet213 into hybrid scaffolds gave rise to almost full inhibition of *E. coli* and *S. aureus*.	Mixed-bacteria-infected wound dressing	[95]
Col (2.5–3 mg/mL) HA (1.5 mg/mL)	Rat tail tendon	Polyelectrolyte multilayers	GTA (8% *w*/*v*)	AMP LL37 (2, 8, 16 µM)	Sustained release of the AMP killed planktonic bacteria.	Broth dilution, bacterial adhesion test, live/dead assay	*E. coli* Primary rat hepatocytes	The incorporation of 16 µM of AMP LL37 showed almost 3% of live bacteria on the scaffold surface.	Antimicrobial coating	[97]
Col PLC (14% *w*/*v*)	Not specified	Membrane (ready-to-use product)	Not available	AMP LL37 (10–40 µM)	Membranes containing different LL-37 concentrations released LL-37 in the same quantity.	Not studied	L929 fibroblasts	Not studied	Collagen membrane for guided bone regeneration	[98]
**Herbal extract-based approaches**										
Col (2% *w*/*v*)	Bovine skin	Membrane	Not available	Propolis NPs (200 µg/mL)	Not studied	Not studied	HDFs	Not studied	Dermal patch	[123]
Col CNC (7 *w*%)	Bovine tendon	Sponge	Not available	Curcumin (5 mg/mL)	99.3% of curcumin was released within the first 24 h.	Disc diffusion	*E. coli*, *S. aureus*, *P. aeruginosa* No in vitro cell culture	Curcumin significantly enhanced the antimicrobial activity of pristine porous scaffolds.	Full-thickness burn dressing	[110]
Col (1% *w*/*w*) Col/Gel microparticles (50, 125, 250 mg)	Bovine	Sponge	GTA (0.02% *v*/*v*)	*Calendula officinalis* extract (1% *v*/*v*)	Incomplete release of the extract was observed within 14 days at pH 5.5 and 7.4.	Not studied	L929 fibroblasts	Not studied	Dermal substitute	[124]
Col	Goat tendon	Aerogel	Wheatgrass (1, 2, 3% *w*/*v*)	Wheatgrass (1, 2, 3% *w*/*v*)	Not studied	Agar diffusion	*E. coli*, *B. subtilis* Swiss 3T6 fibroblasts, HaCaTs	Hybrid aerogels showed smaller inhibition zones than commercial ampicillin discs against *B. subtilis*.	Wound dressing	[111]
Col (10 mg/mL) GSP (25–100 *w*% to Col)	Cowhide trimming waste	Sponge	Chloroform extract of cinnamon bark (14.28% *v*/*v*)	Cinnamon bark powder (2 g)	Not studied	Broth dilution	*B. subtilis*, *S. aureus*, *E. coli* No in vitro cell culture	The addition of cinnamon bark powder led to great inhibition of all tested strains.	Antimicrobial wound dressing	[107]
Col (9 mg/mL)	Type I (Not specified)	Sponge	Not available	Berberine-oleanolic acid (1–5%)	All samples released about 70% of the drug within 1 h.	Filter paper diffusion	*S. aureus*, *E. coli* MG-63 osteosarcoma cells	Gram-positive bacteria were found more sensitive to developed scaffolds than Gram-negative bacteria.	Scaffold for postoperative bacterial bone infection	[125]
Col (1% *w*/*v*) Chi (1% *w*/*v*) Hap (5% *w*/*v*) PCL (20–80 mg/mL) PVA (0.5–3% *w*/*v*)	Bovine tendon	Sponge	GTA (0.1% *v*/*v*)	*Cissus quadrangularis* caged PCL nanoparticles	Cumulatively more than 80% of the extract was released within 21 days.	Not studied	MC3T3-E1 osteoblasts	Not studied	Bone tissue engineering scaffold	[108]
Col (1% *w*/*v*)	Rat tail tendon	Film	Not available	Thymol (0.25–4 mg/cm^2^)	Not studied	Dehydrogenase activity assay, ATP bioluminescence, microbial penetration assay	*S. aureus*, *E. coli*, *P. aeruginosa* Red blood cells	4 mg/cm^2^ of thymol including films indicated almost full inhibition of all tested strains.	Antibacterial film for wound care applications	[109]
Col (11 *w*% middle layers; 10 *w*% inner layers) PCL (10 *w*% outer layers; 11 *w*% middle layer)	Rat tail	Nanofibrous mat	Not available	*Melilotus officinalis* (2, 4, 8% *w/w*)	Not studied	Not studied	L929 fibroblasts	Not studied	Diabetic foot ulcer dressing	[112]
Col Lipid NPs (10:1 *w* ratio Col/Lipid NPs)	Bovine tendon	Sponge	Not available	Curcumin into lipid NPs	The complete release of curcumin-loaded NPs was observed within 25 days.	Not studied	NIH 3T3 fibroblasts, HaCaTs	Not studied	Composite cryostructurate for wound healing	[126]
Col (10 mg/mL) *Annona* polysaccharide (7.5 mg/mL)	Bovine Achilles tendon	Sponge	Chloroform extract of cinnamon bark	Tetrahydrocurcumin microspheres	28.95 ± 1.7% of the drug was released within 12 h from the composite scaffold.	Disc diffusion	*B. subtilis*, *P. aeruginosa*, *S. aureus* NIH 3T3 fibroblasts	Approximately 20 mm and 10 mm inhibition zone diameters were evaluated against *S. aureus* around the positive control and composite scaffold, respectively.	Antimicrobial wound dressing	[127]
Col (60% *v*/*v* in shell) PVA (50% *v*/*v* in core)	Type I (Not specified)	Nanofibrous core–shell mat	Not available	Licorice roots (50% *v*/*v* in core, and 40% *v*/*v* in shell)	Not studied	Disc diffusion	*S. aureus*, *P. aeruginosa* No in vitro cell culture	Bio-nano scaffolds did not show any activity on the inhibition of *P. aeruginosa* growth.	Hybrid bio-nano wound dressing	[128]

## 4. Combination Approaches

The combination of antimicrobial bioactive agents has been studied to increase the treatment efficacy of collagen-based antimicrobial biomaterial therapies in addition to their single use by taking advantage of synergetic effects of different therapeutics. For this purpose, the simultaneous incorporation of herbal extracts, metal oxides, AMPs, antibiotics, growth factors, and other bioactive molecules into antimicrobial collagen scaffolds has been extensively investigated (Table 5). For example, the synergism of 60 mg/mL of lemon balm and dill essential oils enhanced the antimicrobial activity of collagen-based nanofibers on various Gram-positive and Gram-negative bacterial strains and showed in vivo biocompatibility on Swiss adult mouse model without any causative effect [103]. In the literature, the combination of metal oxide NPs and phytochemicals in biomaterial formulation exhibited advanced tissue regeneration and antimicrobial activity. In a study, the administration of silver NPs and silymarin raised the contraction rate of collagen/chitosan bilayer sponges treated wounds on Wistar rats from 55% to almost complete contraction within 10 days with a thin crust appearance [129]. Similarly, 0.5 *w*% curcumin-loaded graphene oxide NP (2 mg/mL)-reinforced sponge dressings accelerated the wound closure of the open wounds in vivo due to the superior anti-inflammatory and antibacterial features of curcumin and graphene oxide [130], while the cumulative effect of silver NPs and plumbagin led to complete healing of open excision wounds on Wistar rats on the 15th post-treatment day as well as a significant bactericidal effect on both Gram-positive and Gram-negative bacteria [131].

In some cases, the application of antibiotics could not prevent the re-growing of antibiotic-resistant bacterial strains. Although vancomycin-loaded collagen hydrogels were effective in reducing bacterial luminescence on luminescent MRSA, which infected in vivo wounds on the first day, re-growing of bacteria was reported on the 2nd post-treatment day. To overcome this problem, collagen-mimetic-peptide-tethered vancomycin was chosen, and complete inhibition of bacterial growth was achieved by their synergetic effect [132]. Apart from this, the combination of antibiotics with growth factors may ameliorate the rate of wound healing. Silver sulfadiazine, and epidermal and basic fibroblast growth factors, including collagen-based multi-layered nanofibers, presented ideal healing for in vivo full-thickness wounds thanks to the slow release of growth factors, neutralizing and anti-growth impact of antibiotics, which supported granulation tissue formation as well normal interactions of collagen fibers and fibroblasts with ECM [133].

**Table 5:** Illustrative examples of collagen antimicrobial scaffolds designed with combination approaches. Abbreviations: Antimicrobial susceptibility testing: AST; Weight: *w*; Volume: *v*; Collagen: Col; Chitosan: Chi; Essential oil: EO; poly(D,L-lactide-co-glycolic acid): PLGA; Polycaprolactone: PCL; Antimicrobial peptide: AMP; Nanoparticle: NP; Ammonia: NH_3_; Graphene oxide: GO; Hydroxy apatite: Hap; Elastin-like peptide: ELP; Epidermal growth factor: EGF; Basic fibroblast growth factor: bFGF; Oxytetracycline hydrochloride: OTC; Doxycycline hydrochloride: DXC; Ciprofloxacin: CP; Tobramycin: TB; N-(3-dimethylaminopropyl)-N-ethylcarbodiimide hydrochloride: EDC; N-hydroxysuccinimide: NHS; Glutaraldehyde: GTA; *Escherichia coli*: *E. coli*; *Staphylococcus aureus*: *S. aureus*; *Enterococcus faecalis*: *E. faecalis*; *Salmonella typhimurium*: *S. typhimurium*; Methicillin-resistant *Staphylococcus aureus*: MRSA; *Pseudomonas aeruginosa*: *P. aeruginosa*; *Bacillus subtilis*: *B. subtilis*; *Proteus vulgaris*: *P. vulgaris*; *Streptococcus sanguinis*: *S. sanguinis*; Spontaneously immortalized human keratinocyte line: HaCaT; Recombinant human bone morphogenetic protein-2: rhBMP-2; Human adipose-derived stem cell: hASC; Human dermal fibroblast: HDF.

Composition	Collagen Source	Scaffold Form	Crosslinking	Therapeutic Agent	Release Profile	AST	Bacterial Strain/ Cell Line	Antibacterial Activity	Hypothetic Material	Refs.
Col hydrolysate (2.66% *w*/*v*) Chi (1.5% *w*/*v*)	Bovine tendon Rabbit skin	Nanofibrous mat	Not available	Lemon balm and Dill EOs (60 mg/mL each, 1:1 ratio)	Not studied	Disc diffusion	*S. aureus*, *E. coli*, *E. faecalis*, *S. typhimurium* No in vitro cell culture	While EOs only did not show efficient antimicrobial activity, EO-including membranes, showed significantly higher activity against tested strains.	Medical wound dressing	[103]
Col (5% *w*/*v*)	Not specified	Sponge	GTA (2.5% *v*/*v*)	AMPs Pac-525 and KSL-W (1.5 mg/mL) into PLGA microspheres	Burst release of AMPs occurred within 2 days in both microspheres and scaffolds.	Oxford cup disc diffusion	*S. aureus*, *E. coli* MC-3T3 fibroblasts	Lower doses of AMPs could not lead to inhibition of *S. aureus* and *E. coli* growth.	Scaffold for infective bone defect repair	[134]
Col (3.5% *w*/*v*) Chi (1.5% *w*/*v*)	Hydrolyzed peptide	Bilayer sponge	GTA (0.025% *v*/*v*)	Silymarin (0.5, 1, 2% *w/w*), and silver NPs (3% *w/w*)	A sustained release of antioxidants was observed over 120 h.	Not studied	Cos-7 fibroblasts	Not studied	Antioxidant and antibacterial wound dressing	[129]
Col	Rat tail tendon	Hydrogel	Incubation of Col solution in saturated NH_3_ chamber	Silver NPs (67, 6.7, 0.67 mg/g), and *Cannabis sativa* oil (0.15 mL)	Only 1.5 g of the silver content is released after 24 h.	Disc diffusion, broth dilution	*S. aureus*, *P. aeruginosa* MDCK epithelial cells	Inhibition zone diameter of 67 mg/g silver-NP-including hydrogels increased from 1.45 to 1.75 cm with the addition of EO.	Wound dressing	[135]
Col (1% *w*/*v*)	Fish scale	Sponge	EDC/NHS (1:2:2 GO:EDC:NHS molar ratio)	Curcumin (0.5 *w*%), and GO NPs (2 mg/mL)	82.5% of loaded curcumin was released within 96 h.	Disc diffusion	*P. aeruginosa*, *S. aureus* NIH-3T3 fibroblasts	The inhibition zone diameters around hybrid scaffolds were evaluated as approximately 16 and 15 mm against *S. aureus*, and *P. aeruginosa*, respectively.	Wound dressing	[130]
Col	Rat tail tendon	Membrane	Curcumin caged silver NPs (10, 20 µM)	Curcumin (20–100 µM) caged silver NPs	Not studied	Broth dilution	*E. coli*, *B. subtilis* HaCaTs	20 µM curcumin-caged silver NPs showed 95% growth inhibition of *E. coli*.	Scaffold for biomedical engineering	[136]
Col (3 mg/mL)	Rat tail tendon	Sponge	Plumbagin (1–5 µM)	Plumbagin (1–5 µM) caged silver NPs	Not studied	Disc diffusion, broth microdilution	*E. coli* *B. subtilis* No in vitro cell culture	Hybrid scaffolds presented better antimicrobial activity against *B. subtilis*.	Wound dressing	[131]
Col (8 *w*%) Hap (0, 5, 15 *w*%)	Type I (Not specified)	Nanofibrous mat	EDC/NHS (4:1 *w* ratio EDC:NHS)	Vancomycin hydrochloride, gentamicin sulfate (10 *w*% total, 1:1 *w* ratio)	High concentrations of vancomycin and gentamicin were released for 21 days.	Disc diffusion	MRSA, *S. epidermidis*, *E. faecalis* SAOS-2 osteosarcoma cells	The synergetic effect of two antibiotics yielded increased inhibition zone diameters on MRSA.	Scaffold for the treatment of prosthetic joint infection	[137]
Col (2, 3, 4 mg/mL) Fibrinogen (1.25 mg/mL) Thrombin (0.156 IU/mL)	Bovine	Hydrogel	Not available	Collagen mimetic peptide tethered vancomycin (1.25 mg/gel) into liposomes (30 µg/gel)	Complete vancomycin release was achieved within 12 h.	Broth dilution	*S. aureus*, MRSA NIH-3T3 fibroblasts	Hybrid hydrogels presented higher antimicrobial activity than pristine hydrogels with less than 10^4^ CFU/wound up to the 9th day.	Scaffold for the MRSA-associated treatment	[132]
Col (1% *w*/*v*)	Fish scale	Sponge	GTA (0.25% *v*/*v*)	Mupirocin (1:1 *w* ratio) and *Macrotyloma uniflorum* extract (10% *v*/*v*)	94% of mupirocin was released within 72 h.	Disc diffusion	*B. subtilis*, *S. aureus*, *P. vulgaris*, *E. coli* NIH-3T3 fibroblasts, HaCaTs	The highest antimicrobial activity of composite dressings was observed on *S. aureus*.	Burn wound dressing	[138]
Col (20 *w*%) PCL Zein (15 *w*% PCL/Zein with various ratios)	Fish	Nanofibrous mat	Not available	Zinc oxide NPs (1 *w*%) and *Aloe vera* (5, 8 *w*%)	Approximately 70% of zinc oxide NPs released within 30 days.	Disc diffusion	*S. aureus*, *E. coli* Human gingival fibroblasts	The combination of zinc oxide NPs with *Aloe vera* increased the growth inhibition rate of both bacteria.	Wound dressing	[139]
Col (0.5% *w*/*v*)	Rat tail	Bilayer sponge	GTA (25% *v*/*v*)	Fibrinogen and silver NPs	50% of the included fibrinogen was released within 5 days.	Zone inhibition	*E. coli* No in vitro cell culture	The one-fold increase in silver NPs concentration did not enhance the antimicrobial activity of scaffolds significantly.	Skin tissue engineering scaffold	[140]
Col (6 mg/mL) Elastin-like peptide (18 mg/mL) (1:3 Col/ELP)	Rat tail tendon	Hydrogel	EDC/NHS	rhBMP-2 (0.005% *w*/*v*) doxycycline hyclate (0.5% *w*/*w*)	Bi-phasic release of doxycycline was observed with an initial burst release followed by a sustained release.	Zone inhibition	*E. coli*, *P. aeruginosa*, *S. sanguinis* hASCs	The developed hydrogels could not exert effective activity against *E. coli*.	Bone regenerative hydrogel	[141]
Col (2 *w*%) PCL (15 *w*%) Chi (2 *w*%) PEO (5 *w*%)	Type I (not specified)	3-layered nanofibrous mat	Not available	Silver sulfadiazine (3 mg/mL), EGF, and bFGF (25 µg/mL each)	Between days 5 and 20, the sustained release was achieved with a cumulative release of about 80%.	Antibiotic tube dilution	*P. aeruginosa*, *S. aureus* HDFs	Minimum inhibitory concentration was evaluated as 15 and 30 µg/mL against *P. aeruginosa* and *S. aureus*, respectively.	Wound dressing	[133]
Col (1% *w*/*v*)	Bovine skin	Sponge	GTA (0–1% *w*/*v*)	OTC (1 g/L) DXC (1 g/L)	About 70% of OTC was released from 0.5% of GTA crosslinked scaffolds within 600 min.	Broth dilution	*E. coli*, *E. faecalis*, *S. aureus* Dermal fibroblasts of mouse cell line	Oxytetracycline led to more inhibition growth of tested bacteria.	Dressing for prevention and treatment of infections at the application site	[142]
Col PVA (1:3 *w/w* PVA/Col)	Bovine tendon	Membrane	Not available	Ciprofloxacin and tobramycin (0.3% *w*/*v* for soaking method, 5% *w*/*w* for mixing method)	CP showed more sustained and controlled release. 95% of CP was released after 48 h.	Microdilution, time-kill assay	*S. aureus*, *E. coli* No in vitro cell culture	The efficacy of membranes to kill the tested bacteria was found independent of their release profile.	Ulcerative keratitis dressing	[143]
Col (4 mg/mL)	Not specified	Sponge	Triphenyl phosphate (10% *v*/*v*)	Mupirocin (50 mg) in 5% *w*/*v* Chi microspheres and *Piper betle* extract (5% *v*/*v*)	More than 50% of both drugs are released at the end of 12 h.	Agar disc diffusion	*E. coli*, *S. aureus* No in vitro cell culture	The combination of two antimicrobials slightly increased the antimicrobial activity against both strains.	Wound dressing	[144]
Col (1.06 mg/mL)	*Rapana venosa*	Sponge	Not available	*Salvia officinalis* extract loaded mesoporous silica NPs (10, 20 mg/mL)	Not studied	Broth microdilution	*P. aeruginosa*, *S. aureus* HaCaTs, Human Mel-Juso skin carcinoma cells	The hybrid scaffolds showed at least a two-fold higher minimum inhibitory concentration for *P. aeruginosa*.	Wound dressing	[145]

## 5. Conclusions

The development of antimicrobial therapeutic strategies has an incremental interest in the literature since microbial infections have threatened human health for many years. Biomaterial-based antimicrobial therapies have been considered an alternative and ideal solution for infection treatment because the incorporation of therapeutic bioactive agents into the biomaterial formulation can lead to their controlled and sustained release as well as a decrease in their off-target influences. Moreover, the combination of biomaterials with these molecules’ benefits enhancement in bioactivity and stability of therapeutics; hence, therapeutic efficacy could be improved. Collagen is a prominent polymer for the designing of antimicrobial scaffolds due to its outstanding biocompatibility, biodegradability, hydrophilicity, remarkable cell-attachment affinity, and mechanical, hemostatic, low-antigenic, and non-cytotoxic properties. This review clearly studies the recent developments in different collagen-based approaches in the treatment of microbial infections using various kinds of bioactive molecules incorporated into collagen-based scaffolds for antimicrobial therapies. It is believed that effective treatment strategies can be developed in the future by discovering alternative and non-toxic, nature-inspired therapeutics and by increasing the functionality of biomaterials with more effective and less toxic crosslinking agents.

## Figures and Tables

**Table 1:** Commercially available collagen-based antibacterial products. Abbreviations: Oxidized regenerated cellulose: ORC; Carboxymethyl cellulose: CMC; Ethylenediamine tetra acetic acid: EDTA; Silver chloride: AgCl; Silver (I) oxide: Ag_2_O.

Brand Name	Company	Composition	Collagen Content (*w*%)	Product Form	Refs.
Promogran Prisma™	3M (Saint Paul, MN, USA)	Collagen, ORC, silver-ORC	55	Pad	[31]
ColActive^®^ Plus Powder Ag	Covalon Technologies (Mississauga, Canada)	Collagen, sodium alginate, CMC, EDTA, AgCl	Not available	Pad	[32]
SeptocollA^®^ E	Biomet (Warsaw, IN, USA)	Collagen fleece, gentamicin salts	Not available	Pad	[33]
DermaCol™	DermaRite (North Bergen, NJ, USA)	Collagen, sodium alginate, CMC, EDTA	Not available	Pad	[34]
DermaCol/Ag™	DermaRite	Collagen, sodium alginate, CMC, EDTA, AgCl	Not available	Pad	[35]
SilvaKollagen^®^	DermaRite	Hydrolyzed collagen, Ag_2_O	Not available	Gel	[36]
Puracol^®^ Plus Ag^+^	Medline (Northfield, IL, USA)	Denatured collagen, CMC, sodium alginate, silver, EDTA	Not available	Pad	[37]
Seeskin^®^ P	Synerheal Pharmaceuticals (Chennai, India)	Collagen	Not available	Powder	[38]
CollaSorb^®^	Hartmann (Heidenheim an der Brenz, Germany)	Collagen, sodium alginate	90	Pad	[39]
Genta-Coll^®^ resorb	Resorba (Nürnberg, Germany)	Collagen, gentamicin sulfate	58.3	Sponge	[40]
Collamycin	Synerheal Pharmaceuticals	Collagen, gentamicin sulfate	Not available	Gel	[41]
GenColl	ColoGenesis (Salem, India)	Collagen, gentamicin sulfate	Not available	Gel	[42]
Colloskin^®^M	ColoGenesis	Collagen	Not available	Pad	[43]
Collofiber-MM	ColoGenesis	Collagen, mupirocin, metronidazole	Not available	Powder	[44]
ColoPlug	ColoGenesis	Collagen	Not available	Sponge	[45]
Diacoll-S™	ColoGenesis	Collagen, gentamicin sulfate	Not available	Sponge	[46]

**Table 2:** Illustrative examples of collagen antimicrobial scaffolds designed with non-pharmacological approaches. Abbreviations: Antimicrobial susceptibility testing: AST; Collagen: Col; Chitosan: Chi; Weight: *w*; Volume: *v*; Spontaneously immortalized, human keratinocyte line: HaCaT; *Staphylococcus aureus*: *S. aureus*; *Escherichia coli*: *E. coli*; *Komagataeibacter xylinus*: *K. xylinus*; *Bacillus subtilis*: *B. subtilis*; *Pseudomonas aeruginosa*: *P. aeruginosa*; Bioactive glass: BG; Glutaraldehyde: GTA; Oxidized bacterial cellulose: OBC; Hyaluronic acid: HA; Tetraethoxysilane: TEOS; Beta-tricalcium phosphate: β-TCP; Bone marrow mesenchymal stem cell: BMSC; Human dermal fibroblast: HDF; Human umbilical vein endothelial cell: HUVEC; Human umbilical cord mesenchymal stem cell: hUCMSC.

Composition	Collagen Source	Scaffold Form	Crosslinking	AST	Bacterial Strain/ Cell Line	Antibacterial Activity	Hypothetic Material	Refs.
Col Chi (2% *w*/*v*)	Bovine tendon	Bilayer sponge/nanofibers	Not available	Disc diffusion	*S. aureus*, *E. coli* No in vitro cell culture	The recovery efficiency of *E. coli* and *S. aureus* from composite matrix was evaluated at 52%, and 36%, respectively.	Chronic wound dressing	[53]
Col (8% *w*/*v*) BG (10:1 Col/BG)	Tilapia skin	Nanofibrous mat	GTA vapor for 24 h	Antibacterial activity assay	*S. aureus* HaCaTs, HDFs, HUVECs	Col/BG dressings led to a significant reduction in *S. aureus* colonies.	Wound dressing	[52]
Col OBC Chi (1:0.9:0.25 *w* ratio of OBC/Col/Chi)	Fish skin	Sponge	Not available	ISO20743-2007 [54]	*E. coli*, *S. aureus*, *K. xylinus* L929 fibroblasts	Developed scaffolds could not completely inhibit the growth of tested bacteria.	Antibacterial hemostatic dressing for internal bleeding control	[48]
Col Chi HA (Various *w* ratios)	Rat tail	Hydrogel	Genipin (2, 10, 20 mM)	Well diffusion	*S. aureus*, *E. coli* MG-63 osteosarcoma cells	Developed hydrogels provided more antibacterial activity against *E. coli*.	Bone tissue engineering scaffold	[55]
Col Chi Alginate (Various *w*%)	Tilapia skin	Sponge	Not available	Agar diffusion	*S. aureus* No in vitro cell culture	Composite sponges did not show an effective inhibitory effect on *S. aureus*.	Cutaneous wound dressing	[56]
Col Chi Gelatin (40:40:20 Col/Chi/Gelatin *w*%)	Priacanthus hamrur skin	Sponge	Not available	Disc diffusion	*S. aureus*, *E. coli* No in vitro cell culture	The addition of chitosan slightly increased the *S. aureus* inhibition.	Antibacterial and antioxidant bio-scaffold	[57]
Collagen hydrolysate Chitosan TEOS (0.5–2% *w*/*v*)	Bovine tendon	Sponge	Not available	Disc diffusion	*B. subtilis*, *S. aureus*, *E. coli*, *P. aeruginosa* NIH 3T3 fibroblasts	Developed sponges did not show any antimicrobial activity against *P. aeruginosa*.	Modern collagen wound dressing against traditional collagen dressings	[49]
Collagen β-TCP (9:1 Col/β-TCP *w* ratio)	Type I (Not specified)	Nanofibrous mat	GTA (25, 50 *v*%)	Turbidimetric method	*E. coli*, *S. aureus* BMSCs	Composite mats displayed a more than two-fold higher inhibition rate against *E. coli*.	Bioactive bone scaffold	[58]
Collagen (2.5, 5, 10 mg/mL) Na-Alginate microspheres (3% *w*/*v*)	Type I (Not specified)	Hydrogel	Not available	Not studied	hUCMSCs	Not studied	Wound dressing	[59]

## Data Availability

Data are contained within the article.
